# Cyanobacteria, cyanotoxins, and their histopathological effects on fish tissues in Fehérvárcsurgó reservoir, Hungary

**DOI:** 10.1007/s10661-021-09324-3

**Published:** 2021-08-06

**Authors:** Damjana Drobac Backović, Nada Tokodi, Zoran Marinović, Jelena Lujić, Tamara Dulić, Snežana B. Simić, Nevena B. Đorđević, Nevena Kitanović, Ilija Šćekić, Béla Urbányi, Jussi Meriluoto, Zorica Svirčev

**Affiliations:** 1 Faculty of Sciences, Department of Biology and Ecology, University of Novi Sad, Trg Dositeja Obradovića 3, 21000 Novi Sad, Serbia; 2 Faculty of Biochemistry, Biophysics and Biotechnology, Laboratory of Metabolomics, Jagiellonian University, Gronostajowa 7, 30387 Krakow, Poland; 3 Department of Aquaculture, Hungarian University of Agriculture and Life Sciences, Páter Károly u. 1, Gödöllő, 2100 Hungary; 4 Department of Biomedical Sciences, Center for Reproductive Genomics, Cornell University, Ithaca, NY USA; 5 Faculty of Science and Engineering, Biochemistry, Åbo Akademi University, Tykistökatu 6 A, 20520 Turku, Finland; 6 Faculty of Science, Department of Biology and Ecology, University of Kragujevac, Radoja Domanovića 12, 34000 Kragujevac, Serbia

**Keywords:** Cyanobacterial blooming, Cyanotoxin encoding genes, Microcystin, Fish histology

## Abstract

Cyanobacteria are important members of lake plankton, but they have the ability to form blooms and produce cyanotoxins and thus cause a number of adverse effects. Freshwater ecosystems around the world have been investigated for the distribution of cyanobacteria and their toxins and the effects they have on the ecosystems. Similar research was performed on the Fehérvárcsurgó reservoir in Hungary during 2018. Cyanobacteria were present and blooming, and the highest abundance was recorded in July (2,822,000 cells/mL). The species present were *Aphanizomenon flos-aquae*, *Microcystis flos-aquae*, *Microcystis wesenbergii*, *Cuspidothrix issatschenkoi*, *Dolichospermum flos-aquae*, and *Snowella litoralis*. In July and September, the microcystin encoding gene *mcyE* and the saxitoxin encoding gene *sxtG* were amplified in the biomass samples. While a low concentration of microcystin-RR was found in one water sample from July, analyses of *Abramis brama* and *Carassius gibelio* caught from the reservoir did not show the presence of the investigated microcystins in the fish tissue. However, several histopathological changes, predominantly in gills and kidneys, were observed in the fish, and the damage was more severe during May and especially July, which coincides with the increase in cyanobacterial biomass during the summer months. Cyanobacteria may thus have adverse effects in this ecosystem.

## Introduction

Cyanobacteria are ancient and ubiquitous organisms that have managed to conquer numerous and diverse ecosystems. It has been estimated that the total global cyanobacterial biomass is in the order of 3 × 10^14^ g of carbon, surpassing a thousand million metric tons (10^15^ g) of wet biomass (Garcia-Pichel et al., 2003). Consequently, toxic metabolic products of cyanobacteria — cyanotoxins — are also common, as it was corroborated in a recent review article by Svirčev et al. (2019). The review reports 1118 recorded identifications of major cyanotoxins in 869 freshwater ecosystems from 66 countries worldwide. In Europe, cyanotoxins were documented in 273 freshwater ecosystems (predominantly in lakes) within 25 countries. Microcystins (MCs) were the most commonly detected cyanotoxins (58%) in these studies (Svirčev et al., 2019).

Cyanotoxins were found to be associated with adverse health effects in humans and animals, and MCs and anatoxins (ATX) were presumed causative agents in the majority of the poisonings (Svirčev et al., 2017, 2019). Poisoning cases were recorded in freshwater ecosystems across Europe: the UK, Belgium, Denmark, Finland, France, Greece, Italy, Netherlands, Norway, Sweden, Switzerland, Spain, Serbia, and Hungary (Svirčev et al., 2019). The risks caused by cyanobacterial blooms and cyanotoxins to human health are real and possibly increasing as cyanobacteria vigorously thrive in a large number of aquatic ecosystems in many countries. The synthesis of monitoring data with toxicological, epidemiological, and health data gives solid evidence of adverse effects caused by cyanobacteria and their toxins (Svirčev et al., 2017).

The objective of the present research was to monitor the presence of cyanobacteria and cyanotoxins, as well as to collect data on their effects, in Fehérvárcsurgó reservoir in Hungary. The reservoir constitutes an important freshwater ecosystem that protects the surrounding area from flooding. Due to mining activities, and the existence of a large number of sandstones in the area, a large amount of wastewater ends up in Fehérvárcsurgó reservoir (Nagy et al., 2014). Also, wastewater from the surrounding settlements, as well as organic and inorganic substances that are washed away from the surrounding agricultural land, reaches the reservoir. In the recent decades, blooms of potentially toxic cyanobacterial species were noted, but a very limited amount of data is available (Törökné, 1999; Törökné et al., 2001). The investigation consisted of qualitative and quantitative analyses of cyanobacterial presence, detection of cyanotoxin encoding genes within the cyanobacterial biomass, detection of cyanotoxins in water and fish tissues, and histopathological studies of fish tissues. Similar studies were conducted in the neighboring country, Serbia, on commercial fishponds and lake Ludaš, by the same group of authors (Drobac et al., 2016; Tokodi et al., 2018, 2020), and recently in Hungary on Lake Balaton and Kis-Balaton Water Protection System (KBWPS) (Marinović et al., 2021). The data arising from this type of research and monitoring at the local, regional, and global scale contribute to assessing the risks and consequences cyanobacteria and cyanotoxins can have on human beings and the environment.

## Materials and methods

### Sampling site

Fehérvárcsurgó reservoir is located north of Gaja River, the largest water flow of East Bakony, a mountainous region north of Lake Balaton, Hungary. The reservoir was formed in 1971 and later used for irrigation purposes of cabbage fields. Nowadays, the reservoir is utilized for flood control, industrial and drinking water abstraction, water quality improvement, irrigation, but mostly for fishing and swimming as a popular holiday resort. Its average water depth is 6 to 8 m, but its deepest points reach up to 15 m. The maximal water surface can be up to 223 ha.

### Sampling of water and fish

The sampling of water and fish from the reservoir (47°17′10.4″N, 18°14′50.6″E) was performed during four separate sampling surveys in the spring (April and May), summer (July), and autumn (September) of 2018. Water (12 L) for qualitative and quantitative analyses of cyanobacteria was collected by sweeping with a plankton net (net frame 25 cm ø, net mesh 23 μm) at a depth of 0.3 m (Marinović et al., 2021; Tokodi et al., 2020). All samples were immediately preserved in 10% neutral-buffered formalin (NBF). About 1.5 to 2 L of reservoir water was collected for cyanotoxin analyses during the investigation in July and September of the same year and frozen until the start of the analysis. Fish sampling was carried out in the center of the reservoir with a standard electrofishing device (Marinović et al., 2021; Tokodi et al., 2020). The fish were sampled during all four sampling surveys and consisted of common bream — *Abramis brama* (Linnaeus 1758) and Prussian carp — *Carassius gibelio* (Bloch 1782) (Table 1) of which the latter was sampled only in July. The following organs were collected and pooled per species for each season for MC analysis: gills, muscles, liver, kidney, spleen, intestines, and gonads (testes and ovaries). Tissue samples were also used for histopathological analyses after they were dissected and fixed in 10% neutral-buffered formalin (NBF).

**Table 1:** Fish species caught in Fehérvárcsurgó reservoir during 2018

Month	Species	Number of individuals	Gender ratio (male/female)	TL (mm)
April	*Abramis brama*	5	3:2	285 ± 58
May	*Abramis brama*	7	5:2	305 ± 51
July	*Carassius gibelio*	5	1:4	112 ± 6
September	*Abramis brama*	1	–	135

### Qualitative and quantitative analyses of cyanobacteria

Cyanobacterial taxa were identified at 100 ×, 400 × magnification in accordance with several taxonomic keys (Komárek, 2013; Komárek & Anagnostidis, 1998, 2005) under the Motic BA310 light microscope equipped with a digital camera (Bresser 9 MP) and Micro Cam Lab software. The quantitative analysis of phytoplankton was made with a Motic AE 2000 inverted microscope using the Utermöhl method (Utermöhl, 1958), expressing data as number of cells per ml (Marinović et al., 2021; Tokodi et al., 2020).

### Cyanotoxin encoding gene analyses

As a part of the same project, cyanotoxin encoding gene analyses were conducted as previously described in Tokodi et al. (2020) and Marinović et al. (2021). A short description of the procedure is presented below.

#### Reference strains for PCR analysis

Reference strains were procured from Finnish Environment Institute (SYKE), Pasteur Culture Collection (PCC), Australian National Algae Culture Collection (CS), and National Institute for Environmental Studies Microbial Culture Collection (NIES): MC producer: PCC7820 (*Microcystis*); CYN producers: CS-506 (*Cylindrospermopsis*); STX producer: CS-537/13 (*Dolichospermum*); and ATX-a producer: *Dolichospermum* 123 (former *Anabaena* 123) (SYKE).

#### Extraction of DNA

Water samples of 100–250 mL (depending on the bloom density) were filtered on 2 µm membrane filters (pore size 2–3 µm), and the filters with biomass (filtrides) were freeze-dried. The genomic DNA from both the freeze-dried filters and reference strains (10 mg samples) were extracted using the DNeasy Plant Mini Kit (QIAGEN, Hilden, Germany) according to the manufacturer’s instructions. Minimal modifications were made to successfully extract DNA from the filtrides (double amount of Buffer AP1, Buffer P3, and RNase A was added to suspend the filters entirely). Homogenization of the filter samples was achieved using zirconia/silica disruption beads (0.5 mm) and subsequent vortexing for 1 min. The success of the extraction was examined with a spectrophotometer (NanoDrop ND-1000, Thermo Scientific). The A_260_/A_280_ ratio varied between 1.22 and 2.04, indicating an adequate amount of extracted DNA in the samples.

#### Qualitative PCR

The presence of MC (*mcyE*), CYN (*cyrJ*), STX (*sxtG*, *sxtS*), and ATX (*anaC*) synthetase genes was assessed by qualitative PCR. The PCR reaction mixtures (20 µL) contained 1 × Phire Reaction Buffer, 0.4 µL Phire II HotStart polymerase (Thermo Scientific), 0.2 mM dNTPs (Thermo Scientific), 0.5 µL forward and reverse primers (Table 2), 2 µL of a template, and sterile deionized water (Marinović et al., 2021; Tokodi et al., 2020). The following protocols were used to run PCRs on a C1000 Touch Thermal Cycler (Bio-Rad): initial denaturation for 30 s at 98 °C; 40 cycles of 5 s at 98 °C, 5 s at 62 °C (cyrJ_F, cyrJ_R, sxtG432_F, sxtG928_R, sxtS205_F, sxtS566_R), or 61 °C (HEPF, HEPR), or 52 °C (anaC-genF, anaC-genR) and 10 s at 72 °C; and a final extension of 1 min at 72 °C. The potential inhibition of PCRs was examined with an exogenous amplification control template, which contained 1 µL:1 µL reference/sample. The following strains were used in the control template: PCC7820 for *mcyE*, CS-506 for *cyrJ*, CS-537/13 for *sxtG* and *sxtS*, and *Dolichospermum* 123 for *anaC*. A 1.5% Top Vision agarose gel (Thermo Scientific) dyed with SYBR® Safe DNA gel stain was used to visualize the PCR products. The Quantity One software (v. 4.6.9) of Gel Doc™ XR (Bio-Rad) was used to document the observed bands.

**Table 2:** List of primers used for qualitative PCR

Gene	Primer	5′–3′ sequence	Reference
*mcyE*	HEPF	TTTGGGGTTAACTTTTTTGGGCATAGTC	Dittman et al. (2017)
	HEPR	AATTCTTGAGGCTGTAAATCGGGTTT	
*cyrJ*	cyrJ_F	TTCTCTCCTTTCCCTATCTCTTTATC	Mazmouz et al. (2010)
	cyrJ_R	GCTACGGTGCTGTACCAAGGGGC	
*sxtG*	sxtG432_F	AATGGCAGATCGCAACCGCTAT	Savela et al. (2015)
	sxtG928_R	ACATTCAACCCTGCCCATTCACT	
*sxtS*	sxtS205_F	GGAGTATTDGCGGGTGACTATGA	Savela et al. (2017)
	sxtS566_R	GGTGGCTACTTGGTATAACTCGCA	
*anaC*	anaC-genF	TCTGGTATTCAGTCCCCTCTAT	Rantala-Ylinen et al. (2011)
	anaC-genR	CCCAATAGCCTGTCATCAA	

### Cyanotoxin analyses

In order to guarantee the comparability of the results with earlier results generated within the same project, cyanotoxin analyses in water and tissues were conducted as previously described in Tokodi et al. (2020) and Marinović et al. (2021). A short description of the procedure is provided below.

#### Preparation of water samples for LC–MS/MS

Depending on the bloom density, 100–250 mL of water samples was filtered (pore size 2–3 µm). The biomass on the filter was freeze-dried and the filtrides placed in glass tubes. The toxin extraction was accomplished by adding 3 mL of 75% MeOH and 15 min of bath ultrasonication. Samples were then further extracted with a Bandelin Sonopuls HD 2070 micro-tip probe sonicator for 1 min (30% pulse and 30% energy). After centrifugation of the extracts for 10 min at 10,000 × *g*, 2 mL of the supernatant was evaporated to dryness (50 ˚C, nitrogen flow) in glass tubes. The sample was redissolved in 200 µL 75% MeOH and in 200 µL H_2_O for the analysis of MC and CYN, respectively. After filtration of the samples (0.2 µm GHP ACRODISC 13 PALL Corporation) into inserts, they were ready for LC–MS/MS analysis.

Partial volumes of the filtrates were concentrated by solid-phase extraction (SPE) on Waters Oasis HLB (30 mg). Elution was performed with 5 mL 90% MeOH, and samples were placed into glass tubes. Evaporation was achieved using nitrogen flow, after which samples were redissolved in 200 µL of 75% MeOH and filtered (0.2 µm GHP ACRODISC 13 PALL Corporation) into inserts for LC–MS/MS analysis. Both fractions of the water samples were combined and presented in results.

#### Preparation of fish tissue samples for LC–MS/MS

Fish liver, gills, intestines, muscle, or whole entrails (if there was not enough material) of *C. gibelio* or *A. brama* from Fehérváricsurgó reservoir were analyzed for MCs by LC–MS/MS. Tissues were homogenized, then freeze-dried, and samples of the same organ of all the individuals of the same species were pooled together. Around 400 mg of freeze-dried fish tissue samples was placed into glass tubes, and 10 mL of 75% MeOH was added for extraction of cyanotoxins overnight. Probe homogenization was performed on ice for 30 s, ultrasonication in a bath sonicator for 15 min, and further extraction with a Bandelin Sonopuls HD 2070 micro-tip probe sonicator for 1 min (30% pulse and 30% energy). After centrifugation for 10 min at 10,000 × *g*, 5 mL of hexane to 10 mL of the obtained supernatants was added. The hexane (lipid) layer was discarded by a glass pipette. The samples were first diluted with water and then concentrated by SPE (Waters Oasis HLB 30 mg) and eluted with 5 mL 90% MeOH. Two milliliters of the samples was evaporated (50 ˚C nitrogen flow) in glass tubes and then redissolved in 200 µL 25% MeOH and filtered (0.2 µm GHP ACRODISC 13 PALL Corporation) into inserts. The fish tissue samples were then ready for LC–MS/MS analysis.

#### LC–MS/MS

Toxin analyses were performed by LC–MS/MS (Marinović et al., 2021; Tokodi et al., 2020). The analytical targets consisted of nine MC variants (MC-dmRR, MC-RR, MC-dmYR, MC-YR, MC-dmLR, MC-LR, MC-LY, MC-LW and MC-LF) and CYN.

### Analyses of fish histopathology

*Cyprinus carpio* carp (6 individuals; 3 males/3 females; TL 320 ± 88 mm) was bred at the Department of Aquaculture, Hungarian University of Agriculture and Life Sciences (Gödöllő, Hungary) and used as control in this study. In preparation of fish tissue samples, the similar methodology as described in Tokodi et al. (2020) and Marinović et al. (2021) was used. In short, all tissue samples collected from different organs (gills, liver, kidney, muscle, spleen, intestine, and gonads) were placed in 10% NBF immediately after dissection, and after at least 3 days of fixation, the samples were processed by standard histological procedures. In addition, gill and muscle samples were decalcified before the histological processing using a 75% RDO Rapid Decalcifier solution (Apex Engineering Products Corporation). For tissue processing, the samples were dehydrated in a graded ethanol series, cleared in xylol and subsequently embedded in paraffin wax blocks. Three 5 µm thin sections per tissue per individual were cut and placed onto glass slides and stained with standard hematoxylin and eosin (H&E) staining procedure. The sections were examined under a microscope (Nikon Eclipse 600) and photographed (QImaging Micro Publisher 3.0 digital camera).

## Results

### Presence of cyanobacterial species in water samples

Six cyanobacterial species were recorded in Fehérvárcsurgó reservoir. The most dominant was *Aphanizomenon flos-aquae* Ralfs ex Bornet & Flahault (Table 3).

**Table 3:** Composition of cyanobacteria from Fehérvárcsurgó reservoir in 2018

Taxon	April	May	July	September
**Cyanobacteria**	**cells/mL**	**cells/mL**	**cells/mL**	**cells/mL**
*Aphanizomenon flos-aquae* Ralfs ex Bornet & Flahault	93,500	970,000	2,124,000	1,110,000
*Cuspidothrix issatschenkoi* (Usachev) Rajaniemi et al	–	75,000	287,600	189,000
*Dolichospermum flos-aquae* (Brébisson ex Bornet & Flahault) Wacklin,.Hoffmann & Komárek	–	4,200	15,200	12,100
*Microcystis flos-aquae* (Wittrock) Kirchner	1,100	158,000	205,700	162,000
*Microcystis wesenbergii* (Komárek) Komárek ex Komárek	10,800	46,000	158,300	154,000
*Snowella litoralis* (Häyrén) Komárek & Hindák	–	–	31,200	11,800
**Σ**	**165,400**	**1,418,600**	**2,822,000**	**1,638,900**

– not present in the sample

### Presence of cyanotoxin encoding genes in biomass samples

**Fig. 1:**
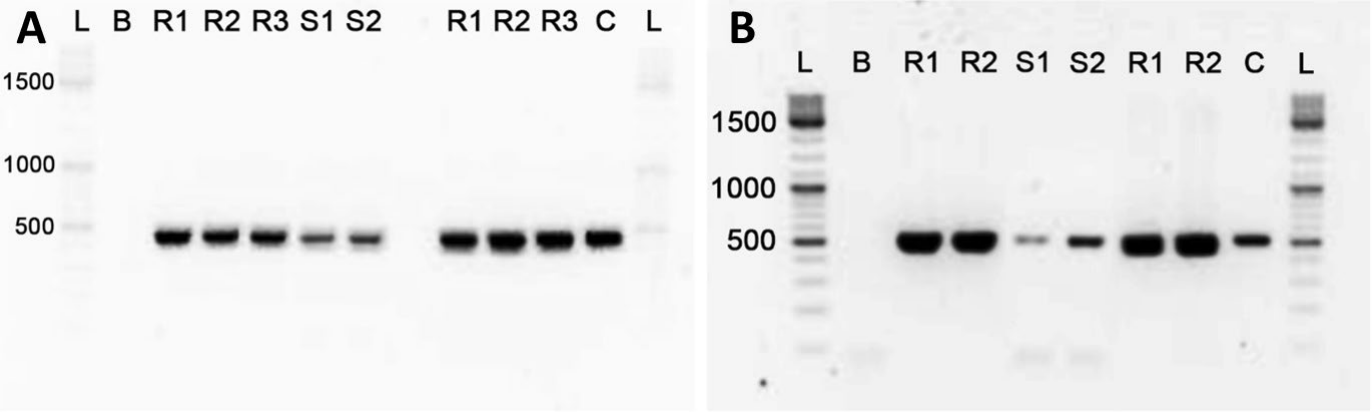
Agarose gel showing presence of cyanotoxin encoding genes in biomass samples, where the *mcyE* gene amplicons (**A**) and *sxtG* gene amplicons (**B**) were observed in July and September 2018 samples from Fehérvárcsurgó reservoir. Legend: R1, R2, R3 — reference strains; S1, S2 — samples from July and September, respectively; B - blank control, C - exogenous amplification control; L - ladder

The MC encoding gene *mcyE* and the STX encoding gene *sxtG* were amplified in biomass samples collected in July and September, while the amplicons of *sxtS* were not observed. The CYN encoding gene *cyrJ* and the ATX-a encoding gene *anaC* were also not amplified in this study (Fig. 1).

### Presence of cyanotoxins in water and fish samples

Cyanobacterial biomass and water samples were tested for the presence of several MC variants and CYN (CYN was analyzed only in the biomass). Only MC-RR was found at a trace concentration (< 0.01 µg/L) in the sample from July 2018. The investigated MC variants were not detected in the tissue samples originating from two fish species sampled in spring, summer, and autumn.

### Histopathological alterations in fish samples

Because of the close taxonomical relation between the common bream and Prussian carp, histopathological results are shown together. Since only one individual was caught during sampling in September 2018, results for this period were not analyzed and presented. The organs of control fish raised at the Department of Aquaculture, Hungarian University of Agriculture and Life Sciences, had a normal histological structure of all the organs.

**Fig. 2:**
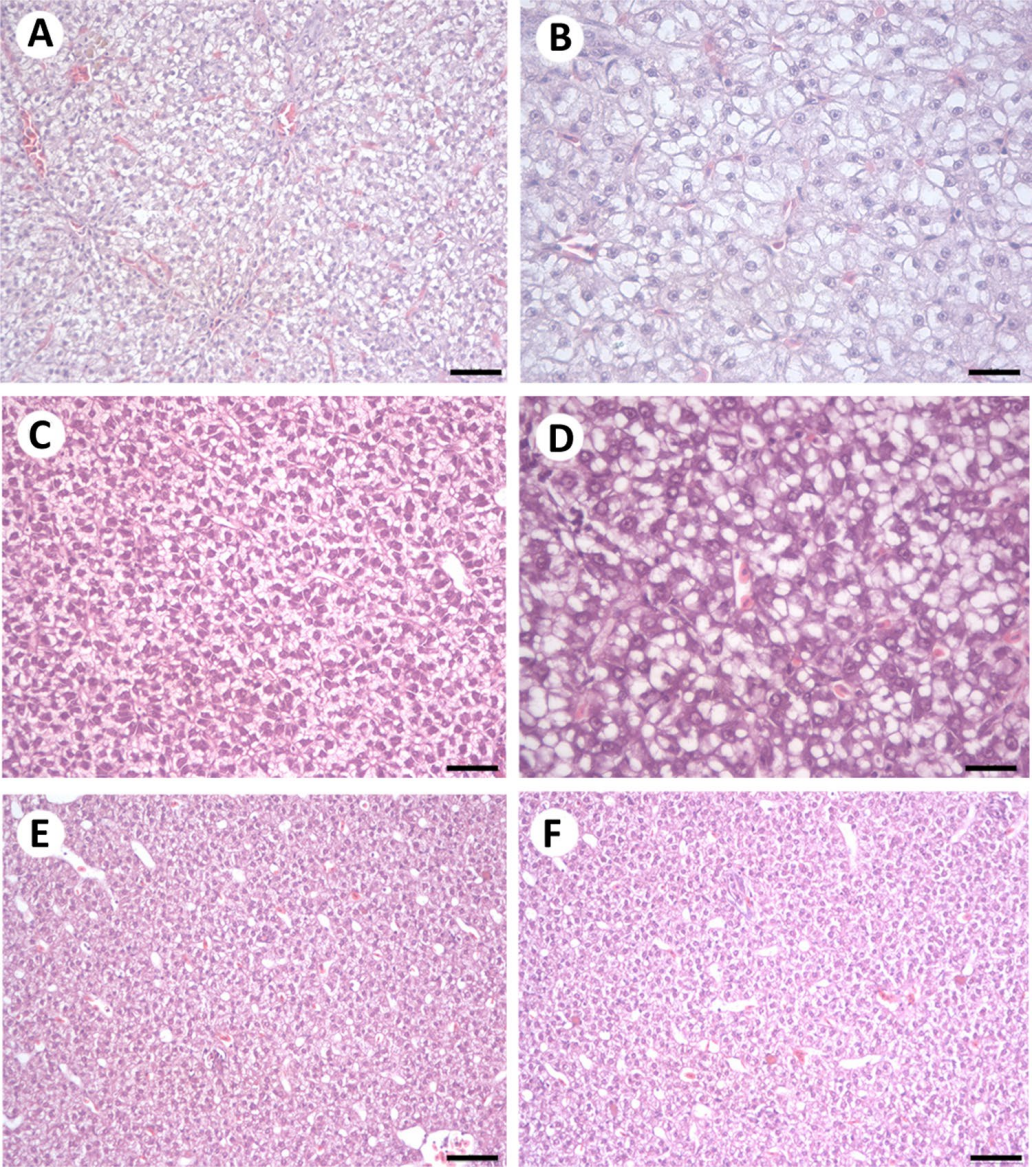
Livers of control individuals (**A**, **B**), as well as of individuals sampled from the Fehérvárcsurgó reservoir during April (**C**, **D**), May (**E**), and July (**F**). (**A**, **B**) Controls displayed normal hepatic structure. (**C**, **D**) Vacuolization of the cytoplasm (microvesicular steatosis). During May (**E**) and July (**F**), no significant histopathological alterations were noted. H&E staining. **A**, **C**, **E**, **F** 50 µm; **B**, **D** 25 µm

Control individuals displayed a normal cord-like parenchyma with polygonal hepatocytes and centrally position nuclei with visible nucleoli (Fig. 2A, B). Most individuals from the Fehérvárcsurgó reservoir displayed normal hepatic structure. The most common alteration was hepatocyte vacuolization to various extents, which was most pronounced during April (Fig. 2C, D). During May (Fig. 2E) and July (Fig. 2F), almost no histopathological changes were observed.

**Fig. 3:**
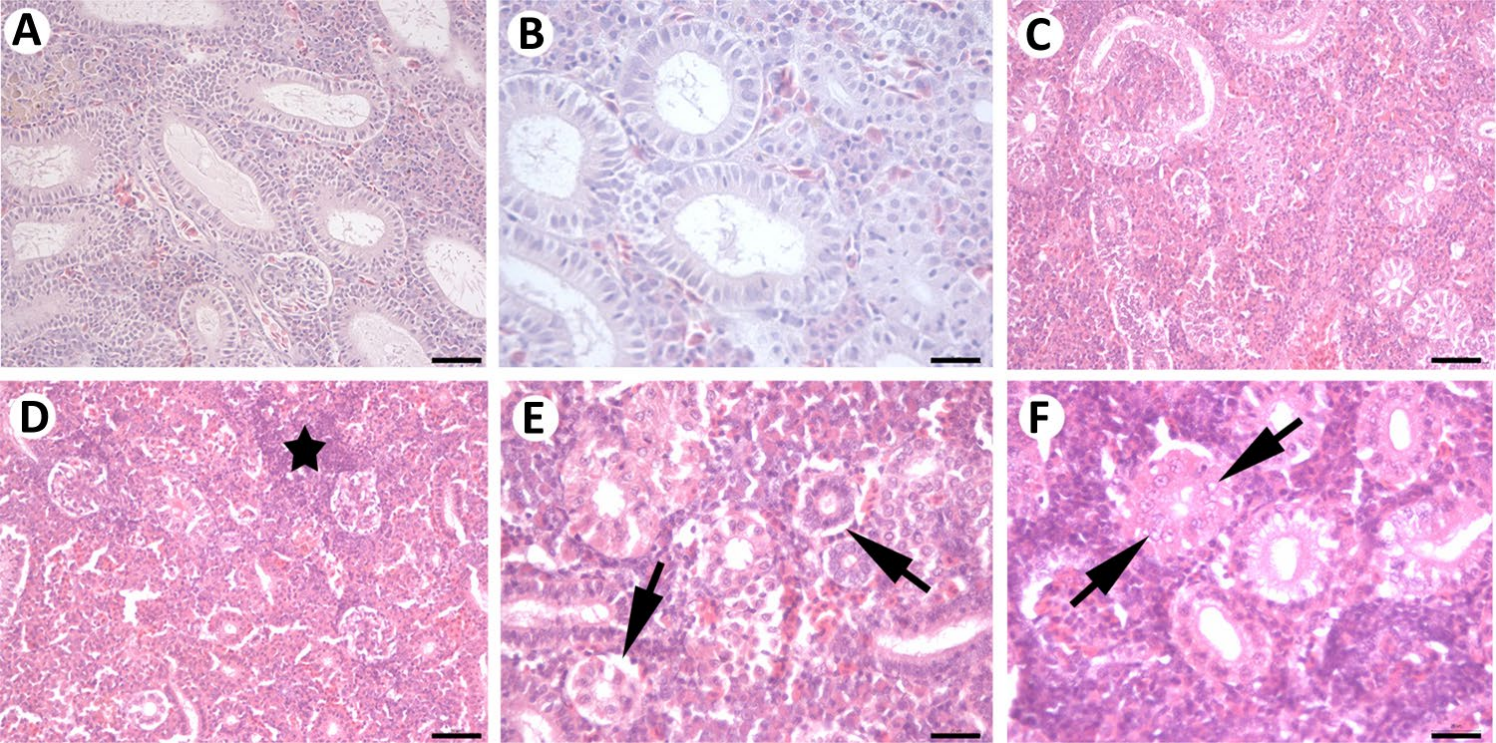
Kidneys of control individuals (**A**, **B**), as well as of individuals sampled from the Fehérvárcsurgó reservoir during April (**C**), May (**D**, **E**) and July (**F**). **A**, **B** Controls displayed a normal renal structure. During April (**C**), we observed vacuolization of tubular epithelial cells, which became much more intense during May (**D**, **E**) and July (**F**). Detachment of the tubular basal lamina (**E**; arrow), lymphocyte infiltration (**D**; star), and karyolysis (**F**; arrow) was also observed. H&E staining. **A**, **C**, **D** 50 µm; **B**, **E**, **F** 25 µm

Kidneys of control individuals displayed normal renal histology (Fig. 3A, B). Individuals from the Fehérvárcsurgó reservoir displayed several alterations throughout the seasons. The most commonly observed alterations were vacuolization of the tubular epithelium (of both proximal and distal tubules: Fig. 3C–F), which led to occasional detachment of the tubular basal lamina (Fig. 3E). Changes within the renal corpuscles were also observed; they involved dilatations of the Bowman’s capsule and mild glomerular atrophy. Lymphocyte and macrophage infiltration (Fig. 3D) was also noted. These changes were the most intense during May and July, when very intense vacuolation of the tubules was detected. However, only mild vacuolations were observed in April. Nuclear damage in the form of karyolysis was also very intense and frequent, mainly during April but also during July (Fig. 3F).

**Fig. 4:**
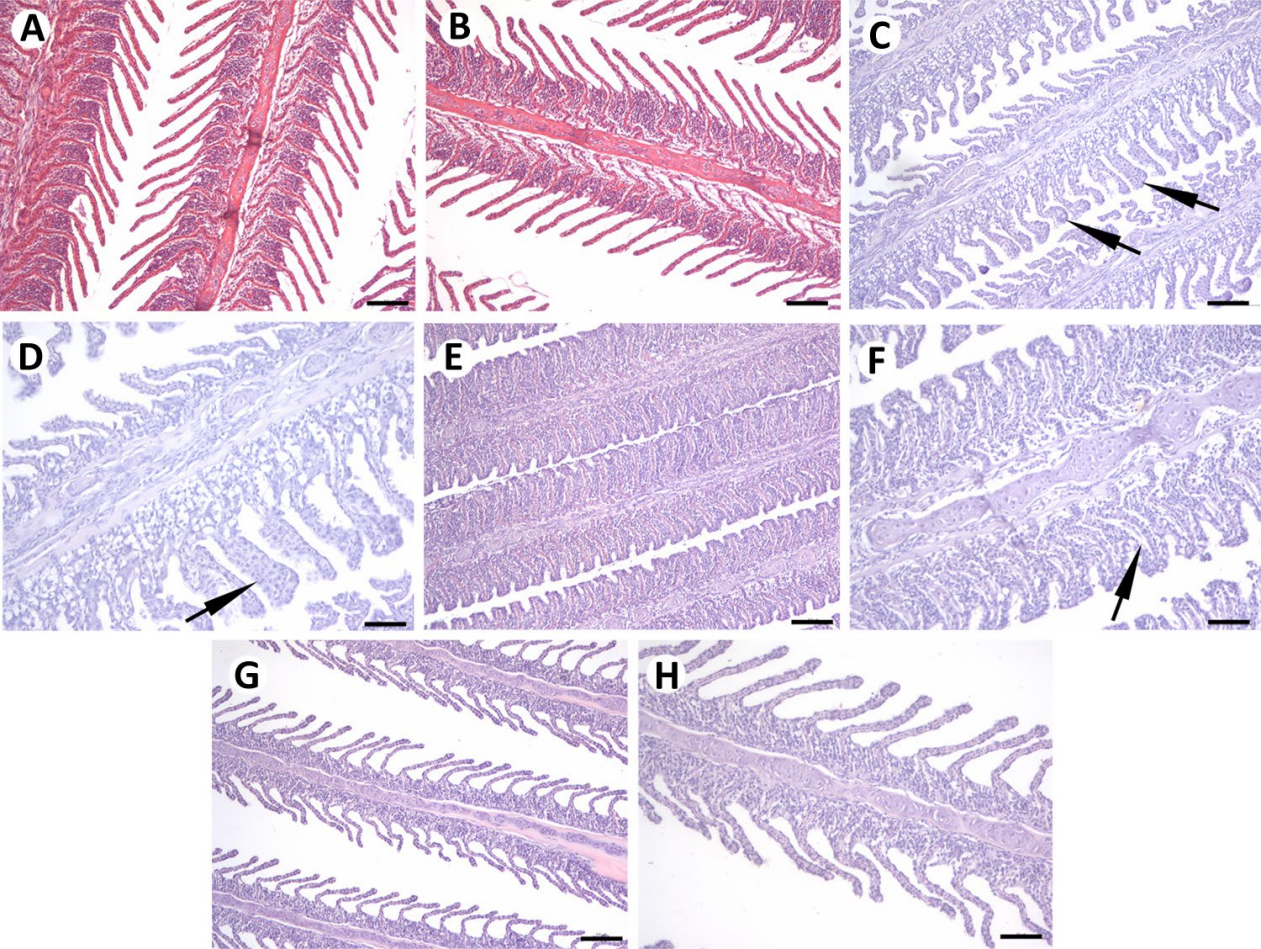
Gills of control individuals (**A**, **B**), as well as of individuals sampled from the Fehérvárcsurgó reservoir during April (**C, D**), May (**E, F**) and July (**G, H**). (**A**, **B)** Controls displayed a normal gill structure. Hypertrophy of epithelium (**C**; arrows), hyperemia (**D**, arrow), and hyperplasia of the lamellar and interlamellar epithelium (**E**, **F**) were observed. During May (**E**, **F**), hyperplasia led to complete fusions of secondary lamellae (**D**), often observed together with epithelial lifting (**F**; arrow). During July (**G**, **H**), no histopathological alterations were noted. H&E staining. **A**, **C**, **E**, **G** 100 µm; **B**, **D**, **F**, **H** 50 µm

Gills of control individuals displayed normal gill histology (Fig. 4A, B). The most frequently observed gill alterations in individuals caught from the Fehérvárcsurgó reservoir were hypertrophy of epithelium (Fig. 4C), clubbing of secondary lamellae, telangiectasia, edema and epithelial lifting, hyperemia (Fig. 4D), and hyperplasia of the lamellar epithelium and the interlamellar cell mass. All mentioned alterations were noted during April. During May, hyperplasia was significantly more pronounced and often led to complete fusions of secondary lamellae (Fig. 4E). Also, in some cases, hyperplasia occurred together with epithelial lifting (Fig. 4F). However, during July, histopathological changes were not recorded, and the gills morphologically looked like those of control fish (Fig. 4G, H).

**Fig. 5:**
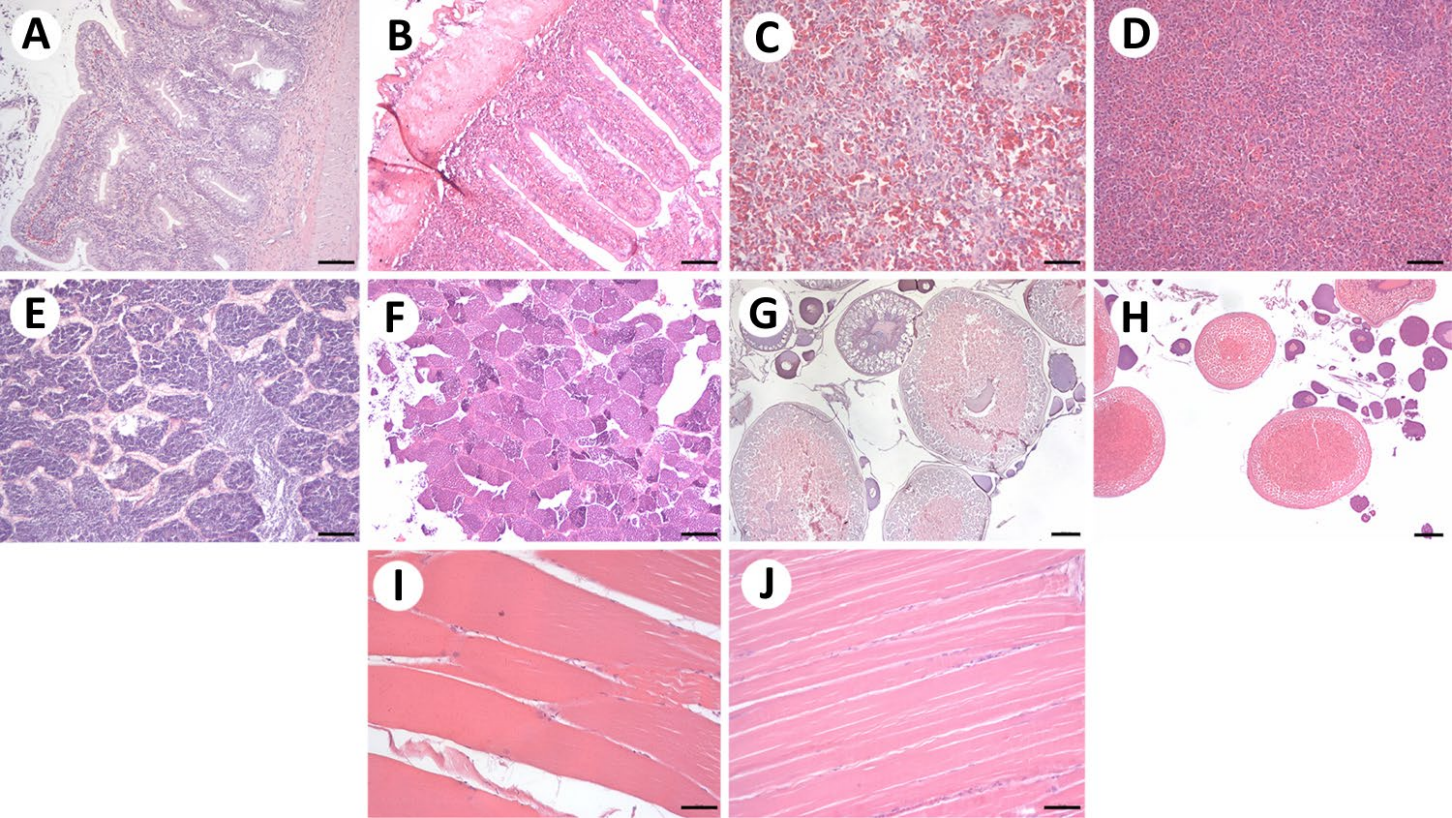
Histopathological sections of intestine (**A**, **B**), spleen (**C**, **D**), testis (**E**, **F**), ovaries (**G**, **H**), and muscles (**I**, **J**) of controls (**A**, **C**, **E**, **G**, **I**) and individuals sampled from the Fehérvárcsurgó reservoir during 2018 (**B**, **D**, **F**, **H**, **J**). H&E staining. **G**, **H** 200 µm; **A**, **B**, **E**, **F** 100 µm; **C**, **D**, **I**, **J** 50 µm

No significant histopathological alterations were observed in the other examined organs of fish from the Fehérvárcsurgó reservoir. The intestines (Fig. 5B), spleen (Fig. 5D), testes (Fig. 5F), ovaries (Fig. 5H), and muscles (Fig. 5J) showed a structure similar to that of the controls (Fig. A, C, E, G, I).

## Discussion

As in every lake, the plankton community in the Fehérvárcsurgó reservoir was changing during the year. During the first sampling in April, three cyanobacterial species were recorded: *Aphanizomenon flos-aquae*, *Microcystis wesenbergii*, and *M. flos-aquae*. The number of cells of the first two species listed exceeded 10,000 cells per mL, which is defined as blooming (Falconer, 1998). The dominant *A. flos-aquae* is one of the most common species of cyanobacteria in freshwater ecosystems. It can produce CYN and STX, while some strains also possess the microcystin synthetase (*mcy*) gene cluster; thus, the production of MCs under some physiological and environmental conditions can occur (Lyon-Colbert et al., 2018). These cyanotoxins can be detrimental to aquatic animals, birds, domestic animals, and humans (Lyon-Colbert et al., 2018). *Microcystis* spp. is a frequently occurring and one of the main bloom-forming genera in eutrophic freshwaters well known for the production of MCs. It is assumed that in Europe, several morphospecies (e.g., *M. aeruginosa*) have a considerably greater portion of MC producers than others (e.g., *M. flos-aquae*) (Via-Ordorika et al., 2004). Although most of the analyzed *M. wesenbergii* strains are non-toxic, several strains were observed to produce MCs (Otsuka et al., 1999; Stoyneva-Gärtner et al., 2021). Thus, the representatives of this species should not be generally considered non-toxic. This morphospecies has a global distribution, including Europe (Jasprica et al., 2005), where it can be found less often than in some other parts of the world such as China (Xu et al., 2008).

In May and July, three further species appeared: *Cuspidothrix issatschenkoi* (synonym: *Aphanizomenon issatschenkoi*), *Dolichospermum flos-aquae* (synonym: *Anabaena flos‐aquae*), and finally *Snowella litoralis*. *C. issatschenkoi* occurs occasionally and uncommonly forms blooms in the northern temperate zone, unlike in the southern temperate zone (Ballot et al., 2018). It is common in Japan, but in Europe, it is an invasive and expanding cyanobacterium (Hodoki et al., 2012). It has a potential to produce ATX-a, homoanatoxin-a, and STXs (as seen in Japan, China, New Zealand, Germany and Portugal), but further knowledge on the worldwide distribution of ATX-a-producing strains of *C. issatschenkoi* is still insubstantial (Ballot et al., 2018; Hodoki et al., 2012). Therefore, it is interesting that a blooming of this species was recorded in the investigated reservoir from May to September 2018. Strains of another observed cyanobacterium, *D. flos-aquae*, have been found to produce ATX-a, ATX-a(S), and MCs in Finland, Japan, Canada, and USA (Bernard et al., 2017). Finally, *S. litoralis* is a cyanobacterium that was not extensively explored. It seems that species belonging to the genera *Snowella* are habitual in lakes of central Europe and Scandinavia, usually at low biomass levels, and they compose a significant portion of cyanobacterial biomass only sporadically (Rajaniemi‐Wacklin et al., 2006). They are often present in late summer and autumn (Rajaniemi‐Wacklin et al., 2006), just as was the case in Fehérvárcsurgó reservoir.

As it was expected in the summer, when the conditions for the blooming of cyanobacteria were the most favorable (higher temperature, light intensity and duration, nutrient enrichment), the number of individual and total cyanobacteria was the highest, with the most dominant species still being *A. flos-aquae*. At the end of the summer, the number of cyanobacteria gradually began to decline. A higher production of cyanotoxins can be expected during favorable growth conditions, and microcystin-RR (MC-RR) was detected in July. While common among MCs, MC-RR is just one of over 279 known variants of MCs (Bouaïcha et al., 2019). Scarce previous studies on Fehérvárcsurgó reservoir have shown that a sample of *M. aeruginosa* collected from the reservoir in September 1995 contained MC-RR (0.17 mg/g), MC-LR (0.38 mg/g), and MC-YR (0.33 mg/g). The sample also caused mortality of *Tamnocephalus* larvae and had a minimal lethal dose of 50 mg/kg in the intraperitoneal mouse assay (Törökné, 1999), induced sensitization in 30% of albino guinea pigs, and provoked slight skin and eye irritation in albino rabbits (Törökné et al., 2001). During our research, the species *M. aeruginosa* was not recorded in Fehérvárcsurgó reservoir.

The potential ability of cyanobacteria in Fehérvárcsurgó reservoir to produce cyanotoxins was supported by the presence of cyanotoxin encoding genes. During July and September, the MC encoding gene *mcyE* and the STX encoding gene *sxtG* were amplified in the biomass samples. The observed amplicons of the *mcyE* gene indicate the presence of MC-producing cyanobacteria in Fehérvárcsurgó reservoir, which is in accordance with the species observed and the obtained LC–MS/MS results. Amplification of the *sxtG* gene was observed in both sampling seasons in Fehérvárcsurgó reservoir, while the amplicons of the *sxtS* gene were not observed in our study. Despite the absence of the *sxtS* gene, the presence of previously reported STX producers *A. flos-aque* (Ferreira et al., 2001) and *C. issatschenkoi* (Li et al., 2003; Pereira et al., 2000) in the reservoir raises concerns over the potential production of STXs. The *sxt* gene cluster is genus-specific and comprises about 30 genes (Mihali et al., 2009), four of which (*sxtA*, *sxtB*, *sxtG*, *sxtS*) are conserved among all STX-producing genera. The presence of all genes within the cluster is necessary for the synthesis of STXs. The observed lack of amplification of the *sxtS* gene may be due to mutations within the gene cluster leading to non-functional gene clusters and an inability of the observed strains to produce STXs.

Several fish tissues were also tested for the presence of MCs but accumulation was not observed. Cyanotoxins could have remained covalently bound to protein phosphatases in the tissue (Ibelings et al., 2005; MacKintosh et al., 1990; Williams et al., 1997a, b), excreted and concentrations reduced by detoxification (Adamovský et al., 2007; Malbrouck & Kestemont, 2006; Snyder et al., 2002; Xie et al., 2004), or concentrations in water were not sufficient enough to be accumulated in detectable levels. Similar conclusions were reached during the research conducted on Lake Ludoš in the neighboring country Serbia during 2018, when cyanotoxins in fish tissue were not detected (Tokodi et al., 2020). However, at the same site a few years earlier (2011/2012), the accumulation of MC-RR and MC-LR in the tissue samples of the Prussian carp from Lake Ludoš was documented, alongside with histopathological changes in liver, kidney, gills, and intestine of exposed fish (Tokodi et al., 2018). Another study from fishponds in Serbia has also reported MC-RR accumulation in muscle tissues and histopathological changes in muscles, gills, intestines, kidneys, and liver of *Cyprinus carpio* farmed in blooming waters (Drobac et al., 2016). Previous studies used a different analytical method (Drobac et al., 2016; Tokodi et al., 2018) better suited for testing accumulation of cyanotoxins in fish samples, which could potentially explain lack of data; however, the method that was used during this investigation was previously successfully used with spiked samples before fish tissue preparation which resulted in positive detection of investigated MCs (Tokodi et al., 2020).

Histopathological changes were also noted in fish caught from Fehérvárcsurgó reservoir. Alterations were more severe during May and especially July, which coincides with the increase in cyanobacterial biomass and the detection of MC-RR in July. However, the absence of hepatic damage indicates that MCs were not the main or only damage-causing agents. Noted vacuolization often occurs due to exposure of fish and mammals to cyanotoxins (Carbis et al., 1996; Drobac et al., 2016; Fischer & Dietrich, 2000; Gupta et al., 2003; Hooser et al., 1990; Jiang et al., 2011; Li et al., 2007; Svirčev et al., 2015); however, this change is very nonspecific and can occur due to the action of various agents, but also due to stress, or dietary imbalance (Thoolen et al., 2010). Also, the alterations in liver observed in April may be the result of cyanobacterial blooms that occurred prior to this study, and it is possible that these changes are accumulations of previous toxin action. The changes observed in the kidneys were more intense than the changes in the liver. Given that Fischer and Dietrich (2000) observed that cyprinid fish display more intense changes in the kidneys rather than the liver after exposure to MCs, and the fact that the kidneys have membrane transporters for MC, it is possible that alterations were caused by MCs. However, since very low concentrations of MCs were detected, it is not clear whether these concentrations are sufficient to cause kidney damage. Most of the mentioned alterations in gills represent defense mechanisms which reduce the respiratory surface and thus reduce the uptake/diffusion of toxic compounds (Lujić et al., 2015). These changes occur after exposure of fish to cyanotoxins (Svirčev et al., 2015) and are most often the result of acute toxin exposure. However, due to the complex relationships that occur within the ecosystem, it is possible that the observed changes are the result of chronic exposure to certain toxins that were not measured in this study but were synthesized by cyanobacteria present in the ecosystem. Since the changes in the liver, kidneys, and gills of fish from this Fehérvárcsurgó reservoir were very mild compared to some other ecosystems (Marinović et al., 2021; Tokodi et al., 2020), and that the cyanobacterial blooming was less intense, the absence of histopathological alterations in the intestines, spleen, testicles, ovaries, and muscles was expected.

Humans can come into contact with cyanobacteria and their metabolites through, e.g., skin and mucosal tissue contact, ingestion of water and food, or aerosol inhalation. Given the multipurpose use of the Fehérvárcsurgó reservoir, fishing, swimming, recreation, and agricultural practices all lead to exposure scenarios. An example from China corroborates the potential threats of MCs in lake/reservoir water: fishermen with chronic and combined exposure to cyanobacteria (contaminated water, fish, and other aquatic organisms from blooming lakes) had serious health problems in the form of hepatocellular damage, and MCs were identified in their serum (Chen et al., 2009). Previously published data indicate the toxic blooming of cyanobacteria in the Fehérvárcsurgó reservoir occurred in the past; however, so far, there is no publication that clearly describes the blooming, production of toxins, or the consequences it has to fish population. Recently, similar investigation was performed in Hungary on Lake Balaton and KBWPS (Marinović et al., 2021). During 2018, blooming of toxic and potentially toxic cyanobacterial species was noted, as well as five MC congeners. Histopathological alterations of the fish from KBWPS were more severe than those described in this study, which could be explained by higher severity of the blooming. Results also showed potential blooming problem throughout the year, as it was the case in this study, which demonstrates necessity of regular monitoring of the cyanobacterial and cyanotoxin distribution, so that potential problems can be anticipated and prevented before escalation.

## Conclusions

Research performed on the Fehérvárcsurgó reservoir in Hungary during 2018 showed that cyanobacteria were present and blooming during sampling in April, May, July, and September. The highest number of cells per mL was recorded in July (2,822,000), and the dominant species was *Aphanizomenon flos-aquae* (2,124,000 cells/mL in July). *Microcystis flos-aquae*, *Microcystis wesenbergii*, *Cuspidothrix issatschenkoi*, *Dolichospermum flos-aquae*, and *Snowella litoralis* were also detected, and their abundance varied depending on the season. Analyses of cyanotoxin encoding genes showed amplification of MC (*mcyE*) and STX (*sxtG*) coding genes, while cyanotoxin analyses indicated a low concentration of MC-RR in July. The identification of MC-RR and cyanotoxin encoding genes indicated that MCs and STXs pose a latent threat that could cause future problems for the reservoir ecosystem and its users, such as fishermen and swimmers. Finally, the analyses of fish tissue did not show the presence of the investigated MCs, but several histopathological alterations, predominantly in gills and kidneys, were observed in fish caught from the reservoir. This type of research can uncover hidden dangers posed by cyanobacteria, and thus give us the advantage in preventing potential consequences and preserving the balance of the ecosystem.

## Data Availability

The data that support the findings of this study are available from the corresponding author, NT, upon reasonable request.
